# Polyphenols in Inner Ear Neurobiology, Health and Disease: From Bench to Clinics

**DOI:** 10.3390/medicina59112045

**Published:** 2023-11-20

**Authors:** Naomi Osakabe, Sergio Modafferi, Maria Laura Ontario, Francesco Rampulla, Vincenzo Zimbone, Maria Rita Migliore, Tilman Fritsch, Ali S. Abdelhameed, Luigi Maiolino, Gabriella Lupo, Carmelina Daniela Anfuso, Elisabetta Genovese, Daniele Monzani, Uwe Wenzel, Edward J. Calabrese, R. Martin Vabulas, Vittorio Calabrese

**Affiliations:** 1 Department of Bioscience and Engineering, Shibaura Institute Technology, Saitama 337-8570, Japan; nao-osa@shibaura-it.ac.jp; 2 Department of Biomedical and Biotechnological Sciences, University of Catania, 95125 Catania, Italy; sergio.modafferi@studium.unict.it (S.M.); marialaura.ontario@ontariosrl.it (M.L.O.); francescorampulla1985@virgilio.it (F.R.); vincenzozimbone@hotmail.it (V.Z.); mariaritamigliore01@gmail.com (M.R.M.); gabriella.lupo@unict.it (G.L.); carmelina.anfuso@unict.it (C.D.A.); 3 NAM Institute, 5020 Salzburg, Austria; tilmanf@hotmail.com; 4 Department of Pharmaceutical Chemistry, College of Pharmacy, King Saud University, Riyadh 11451, Saudi Arabia; asaber@ksu.edu.sa; 5 Department of Medical, Surgical Advanced Technologies “G. F. Ingrassia”, University of Catania, 95125 Catania, Italy; luigi.maiolino@unict.it; 6 Department of Maternal and Child and Adult Medical and Surgical Sciences, University of Modena and Reggio Emilia, 41125 Modena, Italy; elisabetta.genovese@unimore.it; 7 Department of Surgery, Dentistry, Paediatrics and Gynaecology, University of Verona, 37100 Verona, Italy; daniele.monzani@univr.it; 8 Institut für Ernährungswissenschaft, Justus Liebig Universitat Giessen, 35392 Giessen, Germany; 9 Department of Environmental Health Sciences, Morrill I, N344, University of Massachusetts, Amherst, MA 01003, USA; edwardc@schoolph.umass.edu; 10 Charité-Universitätsmedizin Berlin, Institute of Biochemistry, Charitéplatz 1, 10117 Berlin, Germany; martin.vabulas@charite.de

**Keywords:** polyphenols, hormesis, vitagenes, HSPs neuroprotection, Nrf2

## Abstract

There is substantial experimental and clinical interest in providing effective ways to both prevent and slow the onset of hearing loss. Auditory hair cells, which occur along the basilar membrane of the cochlea, often lose functionality due to age-related biological alterations, as well as from exposure to high decibel sounds affecting a diminished/damaged auditory sensitivity. Hearing loss is also seen to take place due to neuronal degeneration before or following hair cell destruction/loss. A strategy is necessary to protect hair cells and XIII cranial/auditory nerve cells prior to injury and throughout aging. Within this context, it was proposed that cochlea neural stem cells may be protected from such aging and environmental/noise insults via the ingestion of protective dietary supplements. Of particular importance is that these studies typically display a hormetic-like biphasic dose–response pattern that prevents the occurrence of auditory cell damage induced by various model chemical toxins, such as cisplatin. Likewise, the hormetic dose–response also enhances the occurrence of cochlear neural cell viability, proliferation, and differentiation. These findings are particularly important since they confirmed a strong dose dependency of the significant beneficial effects (which is biphasic), whilst having a low-dose beneficial response, whereas extensive exposures may become ineffective and/or potentially harmful. According to hormesis, phytochemicals including polyphenols exhibit biphasic dose–response effects activating low-dose antioxidant signaling pathways, resulting in the upregulation of vitagenes, a group of genes involved in preserving cellular homeostasis during stressful conditions. Modulation of the vitagene network through polyphenols increases cellular resilience mechanisms, thus impacting neurological disorder pathophysiology. Here, we aimed to explore polyphenols targeting the NF-E2-related factor 2 (Nrf2) pathway to neuroprotective and therapeutic strategies that can potentially reduce oxidative stress and inflammation, thus preventing auditory hair cell and XIII cranial/auditory nerve cell degeneration. Furthermore, we explored techniques to enhance their bioavailability and efficacy.

## 1. Introduction

There is substantial experimental and clinical interest in providing effective ways to both prevent and slow the onset of hearing loss. Auditory hair cells, which occur along the basilar membrane of the cochlea, often lose functionality due to age-related biological alterations, exposure to high-decibel sounds, or genetic disorders affecting the inner and middle ear. These conditions can manifest as various types of hearing loss, including sensorineural, conductive, or mixed hearing loss, for instance, Pendred or Usher Syndrome [1,2]. Hearing loss is also seen as consequence of neuronal degeneration occurring before or following hair cell destruction/loss. A strategy is therefore necessary to protect hair cells and XIII cranial/auditory nerve cells prior to injury and throughout the aging process.

Within this context, it has been proposed that cochlea neural stem cells may be protected from such age-related and/or environmental/noise insults via the ingestion of protective dietary supplements. In fact, protection of auditory cells was reported with phytochemicals, including ginkgo biloba [3,4,5,6], ginseng [7], and alpha lipoic acid [8]. Of particular importance is that these studies typically display a hormetic-like biphasic dose–response pattern that prevents the occurrence of auditory cell damage induced by various model chemical toxins, such as cisplatin. In these experimental settings the hormetic dose–response also enhances cochlear neural cell viability, proliferation, and differentiation [5]. The cochlear nerve transmits signals from the inner ear to the cochlear nuclei within the brainstem and ultimately to the primary auditory cortex within the temporal lobe. These beneficial activities of the xenobiotics in plants are particularly important since they confirm a strong dose dependency for significant beneficial effects (which is biphasic), whilst having a low-dose beneficial response, whereas extensive exposures may become ineffective and/or potentially harmful. On the other hand, it is known that polyphenols, which were suggested to prevent hearing loss, have very low bioavailability when ingested orally [9] and are extremely unlikely to reach effective high concentrations in hair cells or auditory nerves. Consistently, they are beneficial in preventing hearing loss.

This review explores natural inducers, such as polyphenols, targeting the Nrf2 pathway and its dependent vitagenes, which include also heat shock protein (HSP) system, to develop neuroprotective and therapeutic strategies that can potentially reduce oxidative stress and inflammation, and thus, prevent auditory hair cell and XIII cranial/auditory nerve cell degeneration. Furthermore, we explored techniques to enhance their bioavailability and efficacy.

## 2. Nrf2 and Neuroprotection

**Figure 1:**
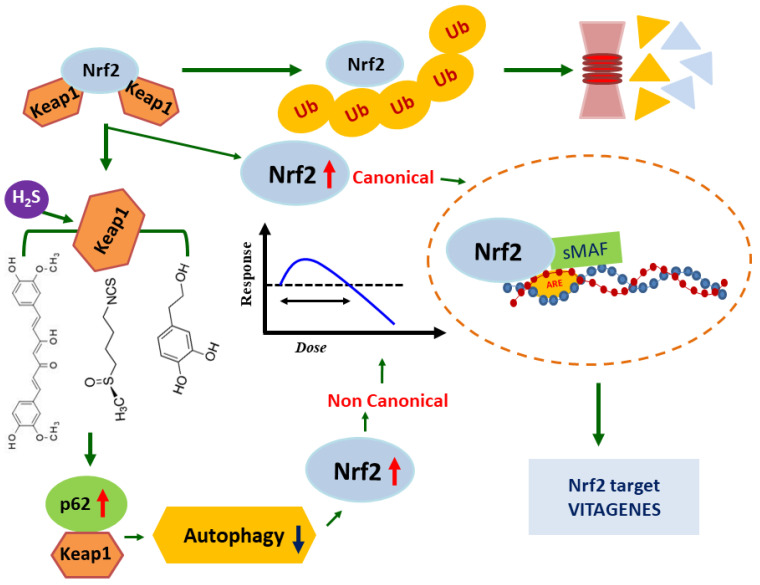
Canonical and non-canonical activation of Nrf2. Nrf2 is localized in the cytosol and interacts with KEAP1 for ubiquitination and proteasomal degradation, under basal conditions. Oxidative stress causes conformational changes of KEAP1-C151, leading to Nrf2 dissociation. Free Nrf2 enters into the nucleus where it forms dimers with MAF proteins and binds to the antioxidant response element (ARE) regulatory sequences of target genes, inducing their expression. In the non-canonical Nrf2 activation, polyphenol-dependent inhibition of autophagy results in increased p62, which competitively binds with KEAP1 and thus contributes to Nrf2 activation in a KEAP1-C151-independent manner. The relationship with hormesis is inferred.

In the central nervous system (CNS), hair cells and XIII cranial/auditory nerve cells, functional redox signaling and low levels of reactive oxygen species (ROS) are thought to play an essential role in neurogenesis and synaptic plasticity [10,11]. These cells possess a low buffering capacity against ROS accumulation, rendering them highly vulnerable to oxidative stress-induced damage, as one major cause of neurodegeneration [12]. NF-E2-related factor 2 (Nrf2) is a master regulator of stress maintenance pathways in several pathological conditions. These include major neurodegenerative diseases such as Alzheimer’s disease (AD) and Parkinson’s disease (PD) [13] along with hearing loss. Under normal circumstances, Nrf2 is located in the cytosol, where its inhibition is modulated by Keap-like ECH-associated protein 1 (KEAP1). Nrf2 is ubiquitously expressed [14] and has a critical role in the defense against toxic insults in the neuron, glial, and astrocytic cells in the brain [15,16]. Specific natural activators of Nrf2 are neuroprotective, making them effective preventive and therapeutic agents for neurodegenerative diseases [17]. In particular, several studies showed that polyphenols have the potential to act as antioxidants, anti-inflammatories, anti-amyloidogenic agents, anti-α-synuclein aggregators, and antidepressants by modulating the Nrf2 pathways and molecular antioxidant biomarkers to prevent the onset and progression of various chronic inflammatory diseases, especially neurodegenerative diseases, both in vitro and in vivo (see Figure 1) [18,19,20,21,22,23,24,25,26].

The activation of Nrf2 is the molecular mechanism by which polyphenols induce neuroprotective effects against oxidative stress and inflammation. Nrf2 accumulates, translocates into the nucleus, and binds to the antioxidant response element (ARE), causing the transcription of multiple target genes, including phase II detoxification enzymes such as NAD(P)H-Quinone oxidoreductase 1 (NQO1), heme oxygenase 1 (HO-1), thioredoxin (Trx), γ-glutamylcysteine synthetase, and glutathione S-transferase (GST). These have been identified as contributors to the antioxidant pathway regulated by Nrf2, protecting against various forms of stress, such as mitochondrial dysfunction, oxidative damage and environmental stress [27,28]. Vitagenes, such as Hsp 70, HO-1, γ-GCs, Trx, and SIRTs were identified by various studies [29,30,31,32,33,34] as biomarkers for stress adaptation, cross-tolerance, and cellular resilience, which are relevant for hormesis or preconditioning [35,36]. Increasing evidence suggests that dysfunction to the Nrf2 pathway significantly contributes to neurodegeneration [37,38,39,40].

**Figure 2:**
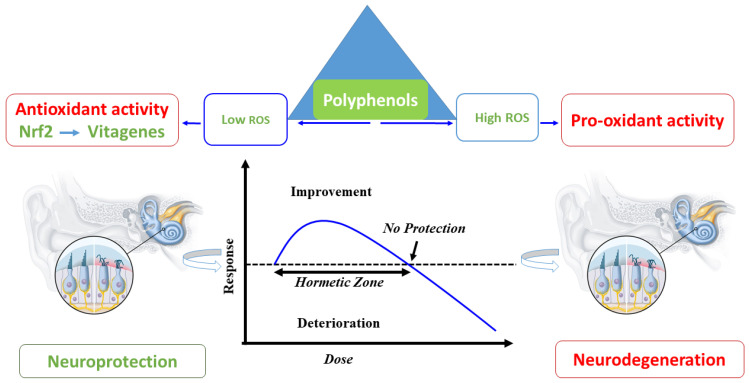
Schematic representation of hormetic neuroprotection. Polyphenols modulate Nrf2-related vitagenes in the low range of hormetic dose–response. On the other hand, at higher stimulation, detrimental effects are observed and neurodegeneration occurs.

Specifically, cellular senescence may contribute to the dysfunction of the Nrf2 pathway and the progression of neuroinflammation and cognitive decline [41]. Furthermore, it is well-documented that Nrf2 is downregulated during times of oxidative stress, inflammation, and neurodegeneration [42,43]. Conversely, the activation of the Nrf2 pathway and related antioxidant vitagenes by polyphenols reduces cognitive decline and neuronal death in animal models of Alzheimer’s disease (AD) and Parkinson’s disease (PD), as well as in humans (Figure 2) [43,44]. Importantly, Nrf2 is crucial for mitochondrial function as it helps to maintain mitochondrial homeostasis, which affects the mitochondrial membrane potential, respiration, oxidative phosphorylation, ATP synthesis, mitochondrial biogenesis and integrity [45,46]. Thus, deregulation of Nrf2 and damage to mitochondria are essential factors causing the development of neurodegenerative diseases since neurons are highly susceptible to oxidative stress [47,48]. Activation of Nrf2 occurs endogenously in response to elevated levels of oxidative stress and inflammation, and can be bursted by nutritional agents administered exogenously. In recent years, researchers have shown interest in the effects of nutritional polyphenols on the prevention and treatment of AD and PD. This was demonstrated by many preclinical and clinical studies focusing on the neuropharmacological activity of these compounds [49,50,51,52,53].

## 3. Heat Shock Response and Neuroprotection

Cellular and organismal functions rely on properly synthesized and folded proteins. The quality of the proteome becomes especially important during disease and the exposure to endogenous and environmental stresses when cells experience physical and chemical fluctuations that they have to adapt to. The homeostasis of protein synthesis, folding, and degradation is called protein homeostasis or proteostasis [54]. Due to its central role in aging and decline of nervous function, proteostasis in neural tissues has become an intense field of research [55].

Many components of the cellular network that ensure protein homeostasis are under the control of the transcription factor Heat shock factor (HSF) 1 and its relatives HSF2, HSF3 and HSF4. This ancient family of gene regulators is involved in the induction of a number of molecular chaperones under stress conditions [56]. Together with Nrf2, HSFs represent a strong defense line against a very broad spectrum of stress inducers and are indispensable components of stress response in human cells. However, the complementarity and interchangeability of the two systems remain less well understood. Specifically, it is not clear which HSF-induced components could replace the Nrf2-induced proteins in protecting the cell from different stressors. HSF1 is the best understood member of the HSF family. In mammalian cells, HSF1 rests in a monomeric inactive state in the cytosol. Upon different stress stimuli, including heat shock, HSF1 trimerizes and acquires DNA-binding capacity [56]. The widely accepted model of HSF1 activation suggests that the transcription factor is kept inactive by association with molecular chaperones. According to the model, the titration of chaperones during stress to other, more affine and abundant targets releases HSF1 for oligomerization [57]. However, oligomerization of HSF1 might not be sufficient for its full activity, and a number of post-translational modifications are known to influence HSF1 function [58]. The known modifications include phosphorylation, acetylation, and sumoylation. Yet another factor to be considered is the role of HSF stability, which in turn affects the cellular levels of the transcription factor. Neurodegeneration is often accompanied by decreased levels of HSF1, which then impairs the capacity of cells to react to stress and induces a protective stress response [58,59,60].

The defects in HSF1 function during aging and neurodegeneration are numerous and are extensively documented [61]. The insufficient activity of HSF1 affects the upregulation of molecular chaperones that otherwise would help protect cellular proteins from misfolding or dissolving already aggregated species. As a consequence, the progression of neurodegeneration is increased, and its clinical manifestations are exaggerated. In a murine model of Huntington’s disease, a lack of HSF1 enhanced mutant huntingtin aggregation [62]. Conversely, overexpression of active HSF1 significantly suppressed huntingtin aggregate formation [63]. Interestingly, HSF2 knock-outs also displayed an increased huntingtin aggregation in the striatum, which caused a reduced lifespan for the experimental animals [64]. Similarly, several Parkinson’s disease models support the protective role of the heat shock response in neurodegeneration. For example, overexpression of a dominant-positive version of HSF1 in human cells decreased a-synuclein levels and ameliorated a-synuclein-induced toxicity [65]. α-Synuclein was found to induce aberrant HSF1 degradation in cell lines and in vivo, which was linked to the activity of the E3 ligase neural precursor cell expressed developmentally downregulated protein (NEDD) 4 that is increased in Parkinson’s [59]. Overexpression of HSF1 in the cerebellum reversed the deficiency of Purkinje cells in Alzheimer’s disease [66], and activation of HSF1 using the HSP90 inhibitor tanespimycin (17-AAG) attenuated Aβ-induced synaptic toxicity and memory impairment [67]. The motor neuron disease amyotrophic lateral sclerosis (ALS) is characterized by aggregates of the superoxide dismutase superoxide dismutase (SOD) 1. Low activity of HSF1 in ALS neurons was linked with their susceptibility to SOD1 aggregation [68]. The neuroprotective effects of HSF1 as exemplified above can be attributed to the induction and action of a number of molecular chaperones. In this regard, HSP70 and HSP90 are the members of the chaperone family that are analyzed most and whose neuroprotective activity is understood best [69]. Chaperones counteract the toxicity of aggregates using at least three mechanisms: (1) preventing amyloid formation, (2) disaggregating misfolded proteins, and (3) sequestering aggregates to specialized subcellular structures [54].

Prevention of amyloid formation is the first strategy of molecular chaperone action. One of the first mechanistic explanations of the protective function of chaperones came with the analyses of huntingtin aggregation in vitro and in vivo. It was shown that HSP70, with the help of its cofactor HSP40, can interfere with the conformational change in mutant huntingtin and thus prevent its aberrant interaction and inactivation of the transcription factor TBP [70]. In these models, induction of HSPs, especially HSP70, is observed in hair cells, accompanied by elevated blood glucocorticoids [71]. It was also reported that in rats, HSP70 (induced by sound conditioning) decreased susceptibility to noise-induced trauma, suggesting the result of enhancement of vitagene through the HSP70/Bmi1–FoxO1–SOD signaling pathway [72].

The neuroprotective effects of HSF1 as exemplified above can be attributed to the induction and action of a number of molecular chaperones. In this regard, HSP70 and HSP90 are the members of the chaperone family, which are analyzed most and whose neuroprotective activity is understood best [69]. Chaperones counteract the toxicity of aggregates by preventing amyloid formation, disaggregating misfolded proteins, and sequestering aggregates to specialized subcellular structures [54]. One of the first mechanistic explanations of the protective function of chaperones came with the analyses of huntingtin aggregation in vitro and in vivo. It was shown that HSP70 with the help of it cofactor HSP40 can interfere with the conformational change in mutant huntingtin and thus prevent its aberrant interaction and inactivation of the transcription factor TBP [73]. The role of the HSP70–HSP40 system in preventing primary nucleation of fibrils and their elongation was demonstrated for different aggregation-prone disease proteins and thus can be considered as the general feature of these molecular chaperones [74,75].

Disaggregation is another mechanism of how chaperones contribute to the neuroprotective effect of the HSF1-mediated stress response. It has been known for a long time that fungi contain the HSP100 family of chaperones that are specialized in dissolving already formed aggregates in an ATP-dependent manner [76,77]. Mammals lack HSP100 chaperones, and their disaggregating function is performed by HSP70 supported by the HSP70 homologue nucleotide exchange factor HSP110 [78,79,80]. Here, proteasomal degradation becomes critical to prevent toxic effects of dissolved species that may exist in potentially dangerous intermediate states [81]. An imbalance of the disaggregating activity of chaperones and the cellular protein degradation capacity would contribute to the paradoxical toxicity of the chaperone overexpression, an example of a complex dose–response relationship in cellular biochemistry.

The third neuroprotective strategy used by cells is to sequester aggregates in dedicated subcellular structures. Molecular chaperones also actively participate in the aggregate sequestration. Specifically, the small Heat shock protein (sHSP) family was shown to be critically involved in this process [82]. Mechanistically, sHSP oligomerization underlies the sHSP-aggregate interactions, which, in contrast to the classical mechanisms of other chaperones, is ATP-independent. In cells exposed to increased temperature, the small heat shock protein sHSP42 concentrates misfolded proteins into small foci, its function mediated by a prion-like domain [83,84,85]. The sequestration activity of sHSPs reduces the substrate burden for other components of the proteostasis machinery and thus contributes to the cellular survival during stress [86]. The concentration of aggregates in mammalian cells lead to the formation of large inclusion bodies, called aggresomes, at the microtubule-organizing center [87]. The sequestration of misfolded species in inclusion bodies has a protective role in a cell. It was shown that inclusion body formation improves survival and leads to decreased levels of mutant huntingtin elsewhere in neurons [88].

In addition to mutant protein-driven aggregation that underlies the pathogenesis of a number of neurodegenerative disorders, there is overwhelming evidence of a general decrease in proteome solubility and an increase in protein aggregation during the aging process, especially in the nervous system. It is believed that the amount of oxidatively damaged proteins increases with age, especially in non-dividing and long-lived cells, such as neurons, which increases the substrate load for the proteostasis machinery probably above manageable levels [54,89,90]. To make things worse, the expression of ATP-dependent molecular chaperones in the aging human brain is repressed significantly [91]. These findings emphasize the need for nutritional and pharmaceutical approaches to boost the heat shock response via HSF1 or support the function of individual chaperones, not only in the clinical setting, but also in the general population to support healthy aging; however, the relationships of molecular chaperones with antioxidative nutrients are complex and not yet well understood. Specifically, polyphenols are known to induce the expression of a number of heat shock proteins and, at the same time, can act as potent inhibitors of their action [92,93]. Furthermore, several polyphenols were reported to increase HSP expression in various organs, reduce oxidative stress, and suppress the exacerbation of symptoms in rodent models of inflammation [94,95]. However, it is still not well understood whether these changes occur in neurons and exhibit a hormetic dose–response, and future research is expected.

## 4. Polyphenols

It is well known that polyphenols are the most diverse of all secondary plant metabolites, with more than 8000 of them identified to date [96], They are divided into subclasses based on their chemical structures, flavonoids (flavonol, flavanol, flavone, anthocyanidin, isoflavonoid, and so on), stilbenes, hydrolyzed tannins, phlorotannins, and phenolic acids. Polyphenols are known to chemically interact strongly with ROS and reactive nitrogen species (RNS) and exert anti-inflammatory effects in vitro and in vivo [97]. Recent studies have reported that some polyphenols reduce damage to cochlear auditory hair cells induced by noise, antibiotics, and anticancer drugs [98,99].

On the other hand, polyphenols are generally less bioavailable [100] and have a limited increase in concentration in the brain. Polyphenol and their associated compound levels can differ significantly in terms of quantity and quality. Clinical studies show that after consuming 10 to 100 mg of a single phenolic compound, plasma concentrations normally do not surpass 1 µM [101]. Aglycones are removed from polyphenol glycosides ingested through food and taken in by the small intestine. It is recognized that the activation of proteins in the excretory system contributes to the low absorption rate from the small intestine. Only a small percentage of these aglycones are taken up by epithelial cells, rapidly conjugated with glucuronide or sulfate, and subsequently secreted into the portal vein. Polyphenols that enter the liver through the portal vein undergo reactions such as glucuronic acid conjugation, sulfate conjugation, methylation, etc. After conversion, they are secreted as metabolites into the peripheral blood [102,103]. These metabolites are then transported into the bloodstream and eliminated in the urine. Part of the polyphenols that reach the lower part of the gastrointestinal tract are degraded by intestinal bacteria and many types of degradation products are produced in the colon [104,105]. The microbiota metabolism of polyphenols involves cleaving glycosidic linkages and breaking down the heterocyclic backbone [106]. It was reported that the primary genera responsible for the metabolism of various phenolics, such as isoflavones, flavonols, flavones, and flavan-3-ols, are *Clostridium* and *Eubacterium*. The microbiome breaks down procyanidins and tannins that remain unabsorbed in the upper gastrointestinal tract and produces characteristic, low-molecular-weight degradation products in the colon [107]. For instance, procyanidins and flavan-3-ol oligomers are examples of compounds that can undergo intestinal bacterial catabolism to produce 3,4-dihydroxyphenylvaleric acid, which is further degraded into phenolic acids like 3,4-dihydroxyphenylpropionic acid and 3,4-dihydroxybenzoic acid [108]. Since these degradation products are more readily absorbed than the original polyphenol compounds, it was suggested that they might impact biological activity.

In addition, recent reports suggest that polyphenol consumption causes temporary changes to the composition of microbiota [107]. These alterations in microbiota in the gut modulate the intestinal barrier function, innate and adaptive immune response, and signaling pathways and then impact host homeostasis [109]. However, only a limited number of papers in relation to this are reported to date, and the relationship between the alterations in the intestinal environment of polyphenols and their positive effects on the central nervous system, cardiovascular system, and inner ear is uncertain. Nevertheless, the mechanism by which polyphenols show preventive or therapeutic effects against inner ear disorders has attracted much attention. Therefore, we selected papers related to polyphenols and hearing loss from Google Scholar, Scopus, PubMed, and the Web of Science using the following keywords in June 2023: “polyphenol, flavonoid, flavanol, flavanon, flavone, flavonol, flavanonol, isoflavon, procyanidin, anthocyanin, chalcone, tannin, phenolc acid, and/or curcumin” and “hearing loss, hair cell, and/or neurodegeneration”. In addition, papers containing mixtures of polyphenols such as plant extracts or whose contents were unknown were excluded from the selection. We additionally examined the literature on the bioavailability of compounds whose effectiveness was expressed in the survey findings.

### 4.1. Flavonoids

**Figure 3:**
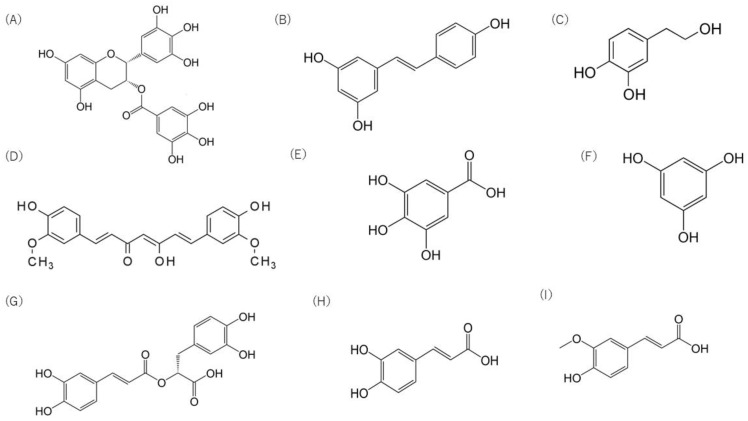
Chemical structure of major polyphenolic compounds: (**A**) epigallocatechin gallate, (**B**) resveratrol, (**C**) hydroxytyrosol, (**D**) curcumin, (**E**) gallic acid, (**F**) phloroglucinol, (**G**) rosmarinic acid, (**H**) caffeic acid, (**I**) ferulic acid.

Flavonoids are a group of compounds characterized by a backbone of 15 carbon atoms and two phenyl rings A and B linked by a heterocyclic (pyran) ring [110]. Epigallocatechin gallate (EGCG, Figure 3A), a flavanol as a subgroup of flavonoids, is the most representative polyphenolic compound found in green tea [111]. The anti-inflammatory activities of EGCG are well demonstrated in in vitro and in vivo models, accompanied by increased Nrf2 signaling and inhibited NFkB pathway through its antioxidant activity [112,113,114]. These effects are not observed in Nrf2 knockout mice or their cells, suggesting that EC indirectly induces Nrf2 and its underlying signals to exert its effect [113]. EGCG was reported to inhibit NO-mediated ototoxicity. Kim et al. examined the preventive effect of EGCG on a HEI-OC1 auditory cells ototoxicity model using RNS generated by S-nitroso-N-acetylpenicillamine (SNAP), an NO donor. SNAP released cytochrome c and activated caspase-3 from the cells, but EGCG inhibited this change and the subsequent reduction in cell viability [115,116,117]. EGCG also reduced SNAP-induced destruction of hair cell arrays in the organ of Corti by suppressing the activation of caspase-3/NF-κB. It was also suggested that EGCG may protect cochlear hair cells from the ototoxic drug gentamicin. Gentamicin is known to increase γ-secrectase activity and to suppress Notch signaling, which inhibits hair cell proliferation. EGCG inhibited γ-secretase activity and promoted hair cell proliferation and regeneration in the damaged cochlea [118]. ROS and RNS in the cochlea, which increase with age, are known to cause age-related hearing loss. It was reported that the redox balance in the cochlea of rats fed for up to 24 months was gradually altered, with decreased SOD and GPx and increased total ROS/RNS and nitrotyrosine. However, supplementation with a mixture of flavonoids and other phenolics consisting of quercetin, rutin and morin, tannic acid, resveratrol, and gallic acid in their drinking water significantly inhibited these changes, thus protecting against ototoxicity [119]. In addition, flavanol derived from cocoa, consisted of (-)-epicatechin and its oligomers, inhibits the activation of senescence-related apoptotic signaling by decreasing oxidative stress in auditory senescent cells such as the HEI-OC1 cell, the stria vascularis-derived cell line SV-k1, and the organ of Corti (OC-k3) cells derived from the auditory organ of a transgenic mouse [120].

EGCG is shown to have poor bioavailability in human volunteers. The peak plasma concentration of EGCG 1.5–2.6 h after intake of green tea is in the submicromolar range and half-lives of it last for a few hours [121]. Moreover, the bioavailability of EGCG is only about 2%. This low bioavailability of EGCG is related to its instability in the intestine, poor pharmacokinetic properties, and tissue accumulation. It is also well known that cocoa flavanols have poor bioavailability [122]. In particular, the oligomers that make up 80% of them are not absorbed at all and can only be detected in small amounts in the blood [123,124]. Despite this low biophysical availability, it is very interesting that they prevent hair cell damage and understanding the mechanisms involved is important.

### 4.2. Stilbene

Stilbene is characterized by a carbon skeleton of 1,2-diphenylethylene (C6–C2–C6), consisting of an ethylene moiety in the middle of two benzene rings [125]. Several plants produce natural stilbenes to protect themselves against stress conditions such as excessive ultraviolet (UV) radiation, heat, insect attack, and fungal or the bacterial infection Pecyna. The neuroactive compounds of blueberry pterostilbene possess the ability to activate cellular resilience pathways Nrf2-dependent scavenging free radicals and inhibiting the NF-κB inflammatory pathway and consequently protecting against oxidative, inflammatory cell damage, and cytotoxicity [126]. Resveratrol (Figure 3B) is a compound with three hydroxyl groups with a stilbene skeleton and is found in fruits such as grapes and grape products, especially red wine. It exhibits health-promoting effects including prevention and/or treatment of neurodegenerative disorders modulating the Nrf2 pathway and stress resilience vitagenes [127]. It was also revealed that resveratrol protects cochlear cells from cisplatin-induced ototoxicity [128] and neuronal cells from hydrogen peroxide-induced oxidative damage dose-dependently [129]. Consistent with hormesis, in vitro studies have shown that a low dose (50 μM) of resveratrol significantly attenuates CoCl2-induced cochlear hair cell damage via activation of Sirt1, which deacetylates NF-κB [130]. Similarly, in vivo studies have demonstrated that a low dose (0.5 mg/kg) of resveratrol exerts otoprotective effects on cisplatin-induced ototoxicity by reducing hearing loss and inflammatory responses (*NF-κB*, *IL6*, and *IL1β*), as well as increasing the expression of antioxidant molecules (*CYP1A1* and RAGE). On the other hand, a high-dose (50 mg) of resveratrol activated pro-inflammatory cytokines and did not ameliorate cisplatin-induced ototoxicity [131]. Moreover, a low-dose of resveratrol inhibited serine/threonine protein kinase (RIPK3)-mediated necroptosis in aging cochlea and delayed the onset of age-related hearing loss in old mice [132]. In addition, resveratrol at a low dose of 10 mg/kg also exerted potent antioxidant effects against amikacin ototoxicity in rats [133]. Notably, intratympanic dexamethasone in synergy with resveratrol provided significant protection against cisplatin-induced ototoxicity in rats [134]. Importantly, resveratrol is the first natural agonist of Sirt1 and induces protection of cochlear hair cells, delaying age-related hearing loss via autophagy. Specifically, the activation of Sirt1 modulates the deacetylation status of ATG9A, which acts as a sensor of endoplasmic reticulum stress and restores cochlear autophagy impairment in C57BL/6 AHL mice [135]. Furthermore, resveratrol upregulated miR-455-5p, reducing apoptosis and oxidative stress in HEI-OC1 cells and inhibiting hair cell damage in cochlear tissues from cisplatin-treated mice via the PTEN-PI3K-Akt signaling pathway [136]. The imbalance of mitophagy and mitochondrial biogenesis is present in the cochlear hair cells during aging or under oxidative stress, contributing to mitochondrial dysfunction and cell damage. In this regard, it was shown that long-term supplementation with resveratrol targeting Sirt1 enhanced mitochondrial function and attenuated spiral ganglion neuron loss in the aging cochlea [137], as well as improving resistance to intense noise exposure damage [138]. Likewise, the pharmacological inhibition of an miR-34a deficiency protected cochlear hair cells and improved age-related hearing loss induced by oxidative stress in C57BL/6 mice [137]. Resveratrol also exhibits promising neuroprotective effects by modulating antioxidant and anti-inflammatory pathways. Other recent evidence showed that resveratrol reduced the phosphorylated and acetylated levels of NF-κB and STAT3, as well as attenuated manganese-induced oxidative stress and inflammatory cytokines by activating Sirt1 signaling. Conversely, EX527, a potent Sirt1 inhibitor, inactivated Sirt1 by inhibiting resveratrol in adult mice [121]. Notably, resveratrol (100 mg/kg) exerted cognitive enhancement and neuroprotection against amyloid and tau pathologies by increasing AMPK protein levels and upregulating the SIRT1 pathway in AD transgenic mice. The improvement of proteostasis by resveratrol, in both healthy and AD mice, suggests that it is a mechanism of brain resilience and defense against neurodegeneration caused by the accumulation of aberrant proteins [122]. Importantly, resveratrol induced significant changes in BBB permeability, edema formation, and the distribution of aquaporin 1 and 4, in addition to the astrocyte profile in the animal model of autism [123]. Taken together, stilbenes, especially resveratrol, induce protection in moderate doses by activating *vitagenes*, primarily targeting the Sirt1 pathway, and this could provide a promising antioxidant therapeutic strategy to delay the neurodegeneration of vulnerable neurons leading to hearing loss and brain dysfunction in humans.

Resveratrol shows high intestinal absorption and rapid and intense metabolism in the gastrointestinal tract and/or liver [139]. Recent stable isotope-labeled studies have reported a high resveratrol absorption rate of 43.9 ± 25.9% [140]. Only resveratrol conjugated derivatives are detected in plasma, such as glucuronide, sulfate, and methoxy forms, with little unchanged form. These resveratrol metabolites might interfere with its therapeutic use because of their difficulty in crossing cell membranes, particularly the BBB, due to their water solubility.

### 4.3. Hydroxytyrosol

Hydroxytyrosol (HT, Figure 3C) is a metabolite of the secoiridoid compound oleuropein found in green olive skins, seeds, and leaves. The degradation of oleuropein to hydroxytyrosol, elenolic acid, and glucose is promoted by acid or heat treatment [141], similar to that in the gastrointestinal tract. In an isotopic study, 90% of ingested HT was absorbed and excreted in the urine within 5 h, suggesting that HT is readily absorbed [142]. The peak of HT in plasma was reported to occur 0.5–1 h after administration. Absorbed HT is oxidized by phase I enzymes to 3,4-dihydroxyphenylacetic acid (DOPAC) and 3,4-dihydroxyphenylacetaldehyde, metabolites produced in the dopamine pathway, which are subsequently metabolized by phase II reactions to O-methylated forms, specifically homovanillic acid [143]. Recent research has focused on the brain health benefits of the major olive oil polyphenols, especially HT and HT-rich aqueous olive pulp extract (Hidrox^®^), which exert multiple preventive and pharmacological activities at low doses, such as antioxidant [144], anti-aging [24], and anti-proliferative effects in vitro and in vivo [145]. Among the phenolic compounds, hydroxytyrosol (50–70%) is the major constituent of the pulp extract, while other polyphenols present include oleuropein (5–10%), tyrosol (0.3%), oleuropein aglycone, and gallic acid [144]. In vitro studies on endothelial cells [146] and the human monocytic cell line [147] have highlighted the ability of HT to downregulate NF-κB activation and its translocation into the nucleus. Recent double-blind randomized preclinical studies have shown that the neuroprotective effect of a HT-enriched diet, in particular a dose of 45 mg HT/kg BW/day, favors recovery after ischemic stroke by improving stroke-associated learning and motor impairments. This effect is probably related to an increase in cerebral blood flow (CBF). In addition, a growing body of evidence reports the brain health benefits of HT supplementation, which at a dose of (50 mg/kg diet) improved cognitive function and reduced Aβ42 and pE3-Aβ plaque in the cortex of TgCRND8 mice [148]. Furthermore, aged mice were shown to have a downregulation of Sirt1, CREB, Gap43 and GPx-1 gene expression in brain tissue [149]. Also in the inner ear, the right dose range is essential to explain the protective effects of antioxidants. Indeed, a high dose of hydroxytyrosol (100 uM) was reported to induce cisplatin-induced ototoxicity in vitro via apoptosis-related JNK and AIF pathways [150].

### 4.4. Curcumin

Curcumin (Figure 3D), diferuloylmethane, is a bright yellow lipophilic pigment found in turmeric [151]. Curcumin is present in the root of the Curcuma longa herb, originally from Asia, in particular from India and Pakistan, a nutritional compound commonly used in food as a spice, mainly in the traditional cuisine of these geographical areas (Middle Eastern and Indian) [152]. The root is the component of greatest nutritional interest, it consists of an aromatic rhizome, yellow or orange in color. Curcuma longa possesses powerful biological activities and has various properties, including antioxidant, anti-inflammatory, antiviral, antibacterial, immunomodulatory, and anticancer activities. It was widely proven since ancient times that this polyphenolic compound has anti-inflammatory characteristics. Due to its anti-inflammatory action, both in the acute and chronic phase, it acts on the arachidonic acid cascade, at the level of cyclooxygenase and lipoxygenase enzymes [153] blocking the synthesis of inflammatory mediators, prostaglandins, and leukotrienes. Curcumin is a strong antioxidant and is one of the compounds expected to be effective against neurodegenerative diseases [154]. In AD model cells, it was reported that curcumin suppresses oxidative stress and increases cell viability by activating the Nrf2 pathway with increased DNA repair enzyme expressions [155]. Curcumin was also shown to improve rotational behavior in rotenone-induced PD rats by reducing oxidative stress via Nrf2 signaling activation. In rats, curcumin alone and/or in synergy with vitamin E prevented cisplatin ototoxicity [156]. In addition, curcumin treatment at a dose of 200 mg attenuated cisplatin-induced ototoxicity and hearing loss by decreasing 4-HNE expression and increasing HO-1 expression in rats [157].

Moreover, dietary curcumin is extensively bio-transformed in phases I and II. A reductase reduces the double bonds of curcumin in enterocytes and hepatocytes to curcumin derivatives [103]. These curcumin derivatives are rapidly absorbed and then found in small amounts in the blood. The plasma concentration ranged from 1 to 3200 ng/mL depending on the dose, which ranged from 2 to 10 g [158]. Studies have shown that curcumin’s bioavailability remains a barrier to understanding the mechanisms behind its neuroprotective effects.

### 4.5. Tannins

Tannins, a class of polyphenolic biomolecules, are large polyphenolic compounds containing sufficient hydroxyl moiety to form strong complexes with various macromolecules. The chemical structures of plant tannins are diverse and can be broadly divided into hydrolyzed tannins and condensed tannins. Hydrolyzed tannins consist of polyphenol nuclei and condensed tannins are oligomeric or polymeric flavan 3-ols with molecular weights ranging from 500 to 20,000 Da [159]. Pomegranate fruit is known to be rich in hydrolyzable tannins, a type of tannin that yields gallic acid on heating with hydrochloric or sulfuric acids (Figure 3E). Administration of pomegranate extract rich in hydrolyzable tannins inhibited cisplatin-induced reduction in distortion product otoacoustic emissions and reduction in mid-turn external ciliated cells in the cochlea [160]. Phlorotannins, the oligomer of phloroglucinol (Figure 3F), are tannins found in brown and red algae. It was reported that dieckol, a phlorotannin, prevents gentamicin-induced hair cell loss in rat cochlear explants [161]. It was also reported that dieckol or phlorofucofuroeckol A, a phlorotannin, suppressed the shift of auditory brainstem response threshold by noise exposure in mice. These phlorotannins showed higher hair cell survival after exposure in the apical turn. In general, highly polymerized, high-molecular-weight tannins are poorly absorbed in the small intestine [162]. The biological activity of tannins was thought to be limited to localized areas such as the gastrointestinal tract so far. Because tannins are a large molecule among polyphenols, ranging from 500 Da to 20 kDa, their bioavailability is known to be extremely low [163]. It is, therefore, unclear whether the efficacy of tannins on the inner ear is due to the breakdown of high-molecular-weight tannins into more bioavailable low-molecular-weight compounds or whether they have an entirely different mechanism.

### 4.6. Phenolic Acid

Rosmarinic acid (RA, Figure 3G is an ester of caffeic acid and 3,4-dihydroxyphenyl lactic acid. It is widely distributed in the Lamiaceae family: basil, lemon balm, rosemary, marjoram, sage, thyme, and peppermint [164]. It was shown to have potent anti-inflammatory properties through antioxidant activities in animal models such as the house dust mite allergy model [165], diesel exhaust particle-induced lung injury model [166], LPS-induced liver damage model [167], or phorbol ester-induced skin irritation model [168], as well as seasonal allergic rhinoconjunctivitis in clinical trials [169,170]. The preventive effect of RA on Cd^2+^-induced ototoxicity in vitro and ex vivo was reported. The results showed that RA inhibited ROS generation, IL-6 and IL-1β production, and caspase-3 translocation in the auditory cells HEI-OC1. RA also prevented the destruction of hair cell arrays in the rat organ of Corti primary explants induced by Cd^2+^ [171]. It was reported that RA inhibits apoptosis in the primary organ of Corti explants. Administration of RA reduced the thresholds of the auditory brainstem response in cisplatin-injected mice, along with inhibiting the caspase-1 downstream signaling pathway, such as activation of caspase-3 and -9, release of cytochrome c, translocation of apoptosis-inducing factor, upregulation of Bax, downregulation of Bcl-2, generation of ROS, and activation of NFκB [172]. Trans-tympanic and systemic administration of RA were previously compared to prevent damage to hair cells caused by noise exposure. Systemic administration of RA to rats, similar to the trans-tympanic treatment, significantly reduced noise-induced hearing loss, and improvement in auditory function paralleled the significant reduction in cochlear oxidative stress, such as O_2_^−^ production and lipid peroxidation, [173]. In addition, RA potentiates the Nrf2/HO-1 signaling pathway, resulting in endogenous antioxidant defenses, as decreased O_2_^−^ production and the expression of 4-HNE, and upregulation of SODs. Finally, RA attenuates noise-induced hearing loss, reducing the threshold shift, and promotes hair cell survival [174]. In rodents and humans, approximately 75% of orally administered RA was reported to be excreted in the urine and detected as metabolites. In addition to methylated RA, the degradation products of RA, caffeic acid (Figure 3H), ferulic acid (Figure 3I), and their conjugates (glucuronide and/or sulfate) were detected in plasma [170,175]. Therefore, it is unclear whether the beneficial effects on hair cells are mediated by RA or its breakdown products, such as caffeic acid or ferulic acid, produced during absorption from the gut and passage through the liver. In fact, caffeic acid, a breakdown product of RA, and its esters were reported to prevent inner ear damage. In streptozotocin-induced ototoxicity, intramuscular administration of caffeic acid ester prevented otoacoustic emissions and the loss of hair cells [176]. Caffeic acid also inhibited hair cell damage induced by cisplatin in vitro [177], or neomycin-induced cell damage in zebrafish [178]. It also prevented HEI-OC1 cell damage by the cisplatin treatment in vitro [179]. Furthermore, noise-induced hearing loss in guinea pigs was significantly reduced by the administration of ferulic acid [180,181]. Based on these findings, it will be necessary to verify the in vivo degradation process of RA to determine the mechanism by which the beneficial effects of RA on inner ear dysfunction occur.

## 5. Attempt to Increase the Bioavailability of Polyphenols

As mentioned above, polyphenols were shown to suppress hair cell damage and potentially reduce the risk of hearing loss. However, one of the limitations to the effectiveness of polyphenols is their poor bioavailability. Therefore, numerous attempts are currently being made to improve the bioavailability of polyphenols. The development of formulations and delivery systems, such as prodrugs or conjugates using nanotechnologies to target the appropriate molecules, has been attempted [182]. For instance, resveratrol-loaded PLGA nanoparticles were observed to enhance resveratrol penetration into the BBB and induce neuroprotection within the brain in experimental models of PD [183]. Of equal importance, the oral administration of novel resveratrol-selenium peptide nanoparticles was reported to effectively improve cognitive dysfunction by interacting with Aβ and decreasing Aβ aggregation and deposition in the hippocampus, reducing Aβ-induced ROS and increasing the activity of antioxidant enzymes in PC12 cells and in vivo. Furthermore, in the same study, resveratrol downregulated Aβ-induced neuroinflammation via the NF-κB/mitogen-activated protein kinase/Akt signaling pathway in BV-2 cells and in vivo and alleviated gut microbiota disturbance, particularly as regards oxidative stress and inflammation-related bacteria such as Alistipes, Helicobacter, Rikenella, Desulfovibrio, and Faecalibaculum [184]. Similar results were also shown in the inner ear disorders via resveratrol-loaded polymer nanoparticles, which did not show any cytotoxicity in vitro and thus could be a suitable model for antioxidant delivery in the cochlea for otoprotection [185]. Nano-encapsulated curcumin administered with dexamethasone protected against cisplatin-induced hearing loss by reducing toxic damage to auditory cells in animal models [186]. Curcumin nanoparticles at a much lower dose than dexamethasone provided otoprotection via the inhibition of Caspase-3 and Bax activation, thereby reducing the concentration of ROS and protecting mitochondrial integrity in hair cells in vitro and in vivo [187]. Interestingly, Yamaguchi and coworkers demonstrated that curcumin abolished intranuclear translocation of nuclear factor-κB-p65 and the generation of 4-hydroxynonenal-adducted proteins found in the cochlea after noise exposure. In particular, Theracurmin^®^, a highly absorbable and bioavailable preparation of curcumin, has shown preventive solid effects on hearing loss induced by repeated noise exposure and suggests it is a promising therapeutic candidate for preventing sensorineural hearing loss [188].

## 6. Development of Model Systems to Elucidate the Hearing Loss Prevention Mechanism of Polyphenols

Clarifying the process by which polyphenols reduce the risk of hearing loss by protecting hair cells is a key priority, as is improving their bioavailability. For this purpose, two model systems are currently considered promising.

### 6.1. C. elegans Models

*C. elegans* is an approximately 1 mm long free-living nematode, which exists predominantly as a self-fertilizing hermaphrodite with a minor percentage of males. It has a rapid life cycle, high reproductive capacity, and limited adult life span of about three weeks under standard culture conditions. The *C. elegans* genome displays about 80% homology to human sequences and more than 42% to human disease-related genes [189]. Functional studies of corresponding or related human genes can be performed either with various mutants available or by RNA interference (RNAi), the latter being easily and exclusively achieved in the nematodes by feeding *Escherichia coli* expressing target-gene dsRNA [190]. Moreover, in the absence of endogenous homologues, *C. elegans* can be transgenically manipulated to express human disease-associated genes in specific cell types, including neurons [191]. Even the co-expression of pathogenic proteins is possible, as was exemplified for the co-expression of β-amyloid and tau, both involved in the pathogenesis of Alzheimer’s disease [192], or for β-amyloid and α-synuclein reflecting the pathogenesis of Lewy-body dementia [193]. Finally, the transparency of *C. elegans* allows the in vivo visualization of neuronal function expression of fluorescent protein reporters in free form or attached to transgenic proteins [194,195]. The primary functional constituents of synaptic transmission found in mammals, including transmitters, receptors, transporters, and ion channels, are preserved in *C. elegans*. Consequently, it could be valuable in assessing hearing impairment.

There are, moreover, *C. elegans* models for further neurodegenerative diseases such as amyotrophic lateral sclerosis, frontotemporal dementia, or Huntington’s disease [196]. Especially for the first, disturbed redox balance appears to represent a pathomechanism. Pan-neuronal expression of the G85R mutant of superoxide dismutase in *C. elegans* is associated with locomotor defects, development of aggregates, and axonal abnormalities [196]. *C. elegans* can be a viable model for neurodevelopmental disorders, depicting challenges in social interaction and communication like autism, and displays great versatility for application.

Like humans, *C. elegans* exhibits a decline in physical ability with age and loss of ability to recover from stress. Those alterations are expressed in the nematodes by reduced body movement and increased sensitivity to heat and oxidative stress [197], These findings suggest that *C. elegans* could be utilized as an advantageous model for evaluating hearing impairment and defects in auditory neurodegeneration. Additionally, it is promising that *C. elegans* is highly responsive to antioxidant nutrients.

Various secondary plant compounds were shown to improve the phenotype in *C. elegans* models of neurodegeneration. In a recent study, the flavonoid chrysin reduced the α-synuclein-induced toxicity in so far as the degeneration of dopaminergic neurons and food-sensing behavioral disabilities, both of which occur after the administration of 6-hydroxydopamine [198]. Chrysin triggered the ubiquitin-like proteasome and superoxide dismutase activities, in agreement with the general concept that oxidative stress and accumulation of non-functional cellular proteins underlie the degeneration of neurons in various neurodegenerative diseases [198]. Hydroxytyrosol from olive oil in its “natural” environment in *C. elegans* Parkinson´s disease models, characterized either by α-synuclein expression in muscles or in dopaminergic neurons, was shown by the authors to significantly improve swimming performance [24]. 10-O-trans-p-Coumaroylcatalpol, a monoterpene extracted from arni, was demonstrated to decrease the aggregation of α-synuclein in transgenic nematodes in association with an increased tolerance against chemical-induced stress, improved chemotaxis index, and reduced content of reactive oxygen species [199]. Moreover, catalpol improved the locomotory ability of aged nematodes, while lipofuscin levels were attenuated, suggesting that catalpol may affect age-associated changes of nematode [200]. At the molecular level, these effects appear to be mediated through DAF-16 and SKN-1, orthologues of mammalian FOXO-transcription factors, and Nrf2, respectively [200].

In a *C. elegans* strain expressing β-amyloid, we demonstrated that the polyphenol quercetin, occurring in substantial amounts in apples and onions, dose-dependently decreased the amount of aggregated proteins in solution and also paralysis [201]. Those effects were mediated through the activation of proteasomal protein degradation or macroautophagy, as discovered when using RNA-interference for members of those pathways [201]. Quercetin, together with kaempferol, were identified as two major effective compounds of Ginkgo biloba extract with regard to the attenuation of basal H_2_O_2_-related reactive oxygen species, which increase in wild-type *C. elegans* with age, but also in a strain with constitutive β-amyloid expression, where initial rates of reactive oxygen species are greatly increased versus the wild-type nematodes [202]. Also, with regard to Alzheimer´s disease, ingredients of olive oil show promising results. Here, it is oleuropein aglycone (OLE), the most abundant polyphenol in extra virgin olive oil, which caused reduced β-amyloid plaque deposition, less abundant toxic β-amyloid oligomers, and decreased paralysis of nematodes expressing β-amyloid in muscle cells [203]. So far, however, investigations of promising compounds from plant food in *C. elegans* models for neurodegeneration are rather scarce.

### 6.2. Organoid Models

The complexity of brain and sensory nerve architecture and physiology, and the scarcity of approaches available, have limited the investigation of their functions over the years. The advent of human-induced pluripotent stem cell (hiPSC) technology and the development of three-dimensional organoid models boosted the opportunities for studying brain function and disorders in in vitro models. hiPSCs are generated by reprogramming adult human cells, obtained from tissues such as skin, blood, or urine, into a pluripotent state [204,205]. The 3D culture system provides the right environment for the stem cells so they can follow their own genetic instructions to self-organize, forming an organ-like tissue composed of multiple cell types. Neural differentiation protocols allow the formation of key features of brain-specific cytoarchitecture and brain processes, including synaptogenesis, differentiation, cell migration, and cell–cell and cell–matrix interactions. This provides researchers with a limitless supply of human organotypic models that recapitulate sophisticated aspects of human in vivo organs, enabling experimental studies that are difficult or impossible to conduct in human subjects [206]. Using brain organoids with these properties has provided a detailed understanding of age-related auditory neuropathy and is valuable for developing therapeutic and preventive strategies for physiologically active substances, such as the polyphenols found in food.

Furthermore, several pathological characteristics of neuropsychiatric disorders were recapitulated using human brain organoids [207]. For example, genetically modified iPSC brain organoids carrying mutations in CHD8 (chromodomain helicase DNA-binding protein 8), one of the most commonly mutated genes in patients with autism spectrum disorder (ASD), bipolar disorder (BPI), schizophrenia and intellectual disabilities, were generated [208,209]. Accordingly, current technology makes it nearly possible to establish a model for hearing organ damage by employing organoids and building an experimental setup capable of assessing active compounds, like polyphenols.

## 7. Polyphenol and the Hormesis Paradigm: Conclusions and Future Perspectives

This review proposes that polyphenols are an effective and clinically applicable way to prevent or delay the onset of hearing loss by activating vitagenes such as Nrf2 and HSP. In addition, these effects, as typified by resveratrol, have potential effectiveness within a hormetic response framework. This conclusion is supported by a limited but consistent series of studies, as summarized below. These studies consist of various experimental protocols, including both direct stimulation and chemoprotective studies within preconditioning protocols. When appropriate dosages were used, the dose–response pattern follows a biphasic dose–response that mirrors the quantitative characteristics of the hormetic dose–response [210]. Additionally, the unified mechanism for hormetic-induced chemoprevention is shown to involve the activation of Nrf2 (Figure 2), as extensively demonstrated in this study. Despite the generally observed hormetic dose–response pattern, it is probable that each studied clinical endpoint has its specific hormetic pattern regarding dose optimality and the quantitative pattern for the stimulatory amplitude and width of the protective treatment zone [211]. However, although there may be variations in the dose–response optimality for specific endpoints, several clinical studies have demonstrated effective dosing for various endpoints at comparable doses. This indicates a potential overlap in the optimal range for multiple therapeutic endpoints, all operating within a comparable hormetic dose–response and mechanistic strategy. These findings may prevent a wide range of neurodegenerative disorders through hormetic mechanisms among diverse groups of people who have varying risk factors for these disorders. This could prove to be an effective public health strategy with widespread implications [212,213,214]. Such a preventive strategy at the population level has the potential to revolutionize public health practices with the realistic objective of considerably reducing the onset and severity of neurodegenerative diseases within society.

Although data from experimental studies look convincing, further well-designed clinical trials are needed to confirm the use of polyphenols for the prevention and treatment of hearing loss, including auditory hair cell and XIII cranial/auditory nerve cell degeneration. Future research could be directed to assessing potential synergy at low doses of the several key active constituents. However, such interactions would likely display a maximum therapeutic response that would to be limited to a 30–60% improvement range of the hormetic dose–response. These suggestive general findings have the potential to guide future studies with respect to the doses employed in the experimental and clinical evaluations. In addition, the employment of polyphenols in the prevention strategies encounters various challenges. Researchers are presently working to increase the preventive effects of polyphenols by improving their bioavailability but further testing is needed to determine whether their efforts will be successful. As previously indicated, there are reports verifying that the ingestion of polyphenols results in significant changes to the intestinal environment. It cannot be excluded that this may have some influence on sensory function. Further research is necessary to comprehend the exact mechanisms that underlie the advantageous impacts of polyphenols.

Overall, the data highlight the neuropharmacological relevance of hormetic nutrition from dietary polyphenols and the upregulation of the stress resilience Nrf2/vitagenes axis as a potential therapeutic target to counteract oxidative stress and inflammation, as well as to prevent neuronal loss associated with neurodegeneration ensuring brain health in humans [215].
